# Clival chordomas and chondrosarcomas in Denmark—Outcomes in 33 patients following the national centralization of treatment in 2010

**DOI:** 10.1007/s00701-024-06241-5

**Published:** 2024-08-29

**Authors:** Mikkel Bundgaard Skotting, Lars Poulsgaard, Jacob Bertram Springborg, Filippa Sundbye, Bodil Elisabeth Engelmann, David Scheie, Urszula Maria Ciochon, Frederikke Guldberg, Kåre Fugleholm

**Affiliations:** 1 Department of Neurosurgery, Copenhagen University Hospital, Copenhagen, Denmark; 2 Department of Oncology, Hospital of Herlev and Gentofte, Herlev, Denmark; 3 Danish Particle Center, Aarhus University Hospital, Aarhus, Denmark; 4 Department of Diagnostic Pathology, Copenhagen University Hospital, Copenhagen, Denmark; 5 Department of Diagnostic Radiology, Copenhagen University Hospital, Copenhagen, Denmark

**Keywords:** Clival, Chordoma, Chondrosarcoma, Adjuvant radiotherapy

## Abstract

**Purpose:**

This 13-year consecutive case series aims to provide a comprehensive overview of all patients operated for clival chordomas and clival chondrosarcomas in Denmark since the centralization of treatment in 2010, comparing outcomes to international series.

**Methods:**

This was a retrospective review of 33 patients with clival tumors, comprising 22 chordomas and 11 chondrosarcomas, who were treated at Copenhagen University Hospital between years 2010 and 2023. Data were collected from digital patient records and pathology reports.

**Results:**

The symptoms leading to diagnosis primarily included double vision, headaches, and dizziness. In general, patients were in good health, with a mean Charlson Comorbidity Index score of 1.6. The complication rate of the index surgery was 51.5%. Adjuvant radiotherapy was applied in 51.5% of the cases. In patients with clival chordomas, the mean age was 51.1 years, ranging from 16 to 83 years. At the time of diagnosis, the mean tumor volume was 20.9 cm^3^ and the five-year overall survival rates were 79.1% (95% confidence interval (CI): 62.4–100). In patients with chondrosarcomas, the mean age was 48.2 years, ranging from 15 to 76 years. At the time of diagnosis, the mean tumor volume was 22.3 cm^3^ and the five-year overall survival 90% (95% CI: 73.2–100).

**Conclusion:**

The centralized treatment of clival tumors in Denmark demonstrates incidence, survival, and complication rates comparable to those found in other international series. Given the variations in treatment strategies, tumor localizations across series, and small sample sizes, the further analysis of larger compiled multicenter datasets for clival tumors could provide more solid evidence regarding the management of these rare tumors.

## Introduction

Clival chordomas and chondrosarcomas are uncommon bone tumors found in the skull base. As these tumors are rare, detailed data on their incidence rates, risk stratification, and prognostic data are limited [2]. Clival chordomas and skull base chondrosarcomas, collectively, have an incidence rate of 0.3 cases per million person-years in the United States [31]. The reported overall five-year survival rates are between 55 and 83% for clival chordomas [10, 11, 14, 21, 24, 29, 30] and between 81.8% [6] and 90% [4] for skull base chondrosarcomas. Both tumor types most commonly occur between the fourth and sixth decades [1, 8]. Despite their distinct origins and prognoses, the two conditions are often considered together due to similarities in location, radiological findings, and clinical presentation [2].

Chordomas are believed to arise from remnants of the primitive notochord [17], whereas chondrosarcomas are thought to originate from mesenchymal cells or the embryonic remnants of the cranial cartilaginous matrix [2]. Compared to chordomas, intracranial chondrosarcomas demonstrate wider anatomical distribution in the cranial region, including spheno-petrosal and spheno-occipital synchondroses, as well as the nasal cavity and paranasal sinuses [23]. Both types of tumors are locally invasive, posing significant management challenges due to their proximity to critical skull base structures, such as the cranial nerves, carotid arteries, and brainstem. These tumors can be diagnosed with magnetic resonance imaging (MRI), and the extent of bone destruction can be assessed using computed tomography (CT) scanning. Diffusion-weighted MRI often aids in the diagnosis [31], and the tumors can be distinguished through immunohistochemistry [13].

In most cases, surgery is the primary relevant treatment, and studies suggest that maximizing resection can reduce the risk of recurrence and improve survival [25, 27]. As mentioned above, achieving complete resection is often difficult due to the invasive nature of the tumors and their proximity to essential skull base structures [7]. Therefore, adjuvant high-dose radiotherapy is often administered [5, 15, 25], and treatment with particles such as protons may result in the better sparing of critical organs at risk as compared to treatment with photons [9, 26, 28].

In Denmark, the treatment for both clival chordoma and clival chondrosarcoma is aggressive surgical excision, meaning that surgery is regarded as the primary treatment modality and gross total resection (GTR) is the primary treatment goal, even if this requires more than one operation (staged surgery). In the case of clival chordoma, radiotherapy is only used when a residual tumor is identified on post-operative MRI following surgery and GTR is no longer realistic or poses an unacceptable risk. In the case of clival chondrosarcomas, radiotherapy is used if the growth of the tumor/remnant is detected, and treatment modality and timing will be less rigorous due to the better prognosis for this tumor as compared to clival chordoma. For the radiation oncologist, treating chordoma and chondrosarcoma tumors presents notable challenges. Due to these tumors’ relative radio resistance, achieving local control necessitates the administration of high total doses of radiation. This requirement, combined with the proximity to essential structures, underscores the need for precise dose delivery and the careful management of treatment-associated risks.

As part of a major structural and administrative reform, the Danish National Board of Health published guidelines for highly specialized regional and national treatment in Neurosurgery. The treatment of clival tumors was specialized at a national level and assigned to the Department of Neurosurgery, Copenhagen University Hospital [20]. Therefore, since the February 26, 2010, we have received all Danish referrals for the treatment of clival tumors. Treatment is currently undertaken in a multidisciplinary collaboration with oncologists at Herlev Hospital and the Danish Center for Particle Therapy, at Aarhus University Hospital.

Few treatment centers have published comprehensive studies regarding outcomes for patients with clival tumors, which makes comparisons and treatment optimization difficult. Therefore, we provide a comprehensive review of all patients in Denmark who have undergone treatment for clival chordomas and clival chondrosarcomas following the national centralization in 2010. To further improve our understanding of the tumors, we provide data on their incidence and outcomes in a national context and, finally, compare these results with international series.

## Methods

We collected a consecutive case series consisting of all clival chordomas and chondrosarcomas managed at the Department of Neurosurgery, Copenhagen University Hospital, Copenhagen, Denmark, over a 13-year period (February 26, 2010, to September 30, 2023). Cases were identified through a comprehensive register of all pathology samples containing chondrosarcoma or chordoma for the specified period at the Department of Pathology. Diagnoses were obtained through histological examination, including immunohistochemical stainings for S100, epithelial membrane antigen (EMA), cytokeratin, and brachyury. Based on the initial MRI scans, only tumors with anatomical localization involving the clivus were included, and clival pathologies other than chordoma or chondrosarcoma, such as metastases, osteosarcoma, and fibrous dysplasia were also excluded. Patients were excluded if our center was not the primary responsible institute for their surgical treatment, except for the calculation of incidence.

The degree of tumor resection was classified into four categories: 1. Gross total resection (GTR): this is defined as the removal of the entire tumor, as determined by post-operative magnetic resonance imaging; 2. Subtotal resection (STR): in this category, roughly 10% or less of the pre-operative tumor mass is still visible on the post-operative control scan; 3. Partial resection: this refers to cases where roughly 10% or more of the tumor remains after the surgical procedure; and 4. Biopsy: this involves the removal of a small tissue sample from the tumor for diagnostic purposes, without attempting to remove the tumor itself.

Relevant data were retrieved from the digital record system at our center and recorded in a secure Redcap database. The amassed data included demographic data; the Charlson Comorbidity Index at diagnosis, the radiological features of the tumor, including volume; histology; presenting symptoms; surgical details; the use of radiotherapy, complications related to treatment, and survival.

We employed Kaplan–Meier analysis to assess overall survival and progression-free survival, where all deaths were included. In addition, we performed competing risks analysis using the cumulative incidence function (CIF) to further characterize the progression rate of the tumors. In this analysis, radiological progression and deaths that were certainly caused by the tumors were included as progression. All deaths with an uncertain cause were considered competing events. All analyses were made using the R software package (Rstudio). Patient inclusion spanned 13 years, with right-sided censoring applied to address shorter follow-up periods resulting from late entry. The estimation of five-year overall survival rates was derived from the Kaplan–Meier plot. Progression was defined as radiological progression. As a radiological criterion of tumor progression, we employed an increase in tumor axes by minimum 2 mm between CT or MRI scans and/or an increase in the axes of malignant osseous destruction by minimum 2 mm between CT scans.

Because the locations of the two types of tumors are similar, the symptoms derived from the tumor and the complication rates for surgical resection are similar. Thus, we have chosen to group the symptoms that led to diagnosis and the post-operative complications for the two tumor types, whereas the treatment modality and survival rates are specified for each tumor type individually. To assess whether surgeons' routine could influence outcomes, a Fisher's Exact Test was used to compare the complication rates of the index operation of patients operated during the first half of the study period with those operated during the latter half.

## Results

From 2010 to 2023 a total of 36 patients were treated for clival chordoma or clival chondrosarcoma at our center. Two patients were excluded because their primary operation occurred before 2010. One patient, a clival chondrosarcoma, was included in the calculation of incidence, but was excluded in the remaining analyses because most of the treatment took place at a center abroad*.* A total of 33 patients were included in the study, comprising 22 (66.7%) patients with chordomas and 11 (33.3%) patients with chondrosarcomas. In general, the patients were in good health prior to their diagnosis and subsequent treatment, with a mean Charlson Comorbidity Index score of 1.6. The mean age of all patients was 49.7 years at the time of diagnosis, with values ranging from 15 to 83 years. Surgical resection was performed in all but one patient, for whom only a biopsy was performed.

A total of 18 (54.5%) patients underwent multiple operations. During the study period, all patients were considered for adjuvant radiation therapy, and 17 (51.5%) patients received adjuvant radiotherapy.

Further information regarding age, incidence, and five-year overall survival is presented in Table 1.

**Table 1:** Incidence, age at initial surgery, and five-year overall survival rate for clival chordoma and chondrosarcoma in Denmark from 2010 to 2023

	Number of patients	Incidence [per million person-years]	Mean age at initial operation (years)	Percentage treated with radiotherapy	Five-year overall survival rate (%)
Chordoma	22	0.3	51.1	63.6	79.1
Chondrosarcoma	11	0.16*	48.2	27.3	90

^*^The patient who was excluded due to receiving most of his treatment abroad has been included in the calculation of incidence [per million person-years]

### Symptoms at the time of diagnosis

In 32 patients, symptoms prompted the decision to conduct a cerebral MRI. The last patient had an incidental finding. At the time of diagnosis, most patients exhibited multiple symptoms. Double vision was the most frequently reported symptom (19 patients), followed by headache (11 patients) and dizziness (nine patients). As for objective neurological deficits, sixth-nerve palsy was the most prevalent (eight patients), followed by trigeminal nerve affection (six patients) and weakness of the extremities (four patients).

### Treatment modalities and survival rate for clival chordomas

A detailed overview of the treatments and outcomes for patients with chordoma can be found in Table 2. In the case of chordomas, the mean age was 51.1 years, with values ranging from 16 to 83 years. At the time of diagnosis, the mean tumor volume was 20.9 cm^3^. The chordomas were histologically graded as classical in 20 (90.1%) cases, while the remaining two were graded as chondroid. All patients underwent surgical intervention. The trans-nasal approach was the favored approach for the index operation and was applied in 14 (63.6%) of the cases. Among the 14 patients who underwent an index trans-nasal operation, one patient (7.1%) experienced cerebrospinal fluid leak as a complication. Following the initial surgery, gross total resection (GTR) was achieved in six patients (27.3%), or seven patients (31.8%) when including patients who underwent staged surgeries. STR was archived in eight patients (36.3%), partial resection in seven patients (31.8%), and a biopsy was performed in one patient (4.5%).

**Table 2:** Treatment and outcome summary for patients with clival chordomas

ID	Sex	Age at radiological diagnosis (years)	Chordoma Grade^20^	Tumor volume on last pre-operative scan (cm^3^)	Surgical approach	Resection grade at index operation	Adjuvent radiotherapy	Number of surgical procedures	Alive at the time of data collection	Observation time (years)
5	Male	65	Classical	15.0	OZ	Partial	No	1	No	0.2
6	Female	21	Chondroid	11.6	Subtemporal	GTR	No	1	Yes	6.4
8	Female	15	Classical	7.2	Far lateral	Partial	No	2*	Yes	7.8
10	Female	22	Classical	21.0	Far lateral	GTR	No	1	Yes	7.7
12	Female	49	Chondroid	2.9	Trans-nasal	GTR	Yes	1	Yes	9.5
14	Male	35	Classical	19.0	Trans-nasal	STR	Yes	4	Yes	8.0
15	Female	60	Classical	3.3	Trans-nasal	STR	Yes	3	Yes	6.0
16	Female	50	Classical	35.2	Trans-petrous	Partial	No	1	No	1.9
20	Male	40	Classical	22.5	Far lateral	GTR	Yes	5	Yes	3.0
21	Female	51	Classical	21.8	Subtemporal	Partial	Yes	2	No	5.6
22	Male	54	Classical	16.7	Trans-nasal	Partial	Yes	1	Yes	10.5
23	Male	54	Classical	38.9	Trans-nasal	STR	Yes	2	Yes	5.8
24	Male	61	Classical	19.5	Trans-nasal	STR	Yes	1	No	3.0
25	Male	64	Classical	21.0	Subtemporal	STR	Yes	2	No	5.8
27	Male	83	Classical	20.8	Trans-nasal	Biopsy	No	1	No	1.5
28	Female	54	Classical	1.9	Trans-nasal	GTR	No	1	Yes	10.4
30	Male	36	Classical	76.0	Trans-nasal	STR	Yes	1	Yes	4.3
31	Male	34	Classical	59.7	Trans-nasal	STR	Yes	2*	Yes	1.9
32	Male	80	Classical	6.3	Trans-nasal	STR	Yes	2	Yes	2.6
33	Male	70	Classical	21.1	Trans-nasal	Partial	Yes	1	Yes	2.0
36	Female	68	Classical	12.6	Trans-nasal	Partial	Yes	1	Yes	1.7
37	Male	59	Classical	6.0	Trans-nasal	GTR	No	1	Yes	1.5

*Abbreviations*: *OZ* Orbitozygomatic approach, *STR* subtotal resection, *GTR* gross total resection

^*^ = Staged surgery

Nine (40.9%) of the 22 patients underwent multiple surgical procedures. Of these, three patients received staged operations. The staged operations include all patients who underwent an additional surgical tumor removal within the first three months after the index surgery.

As mentioned above, radiotherapy is typically used when a residual tumor is identified on a post-operative MRI following the index surgery. Three patients met these criteria but did not receive radiotherapy. One patient died before the initiation of radiotherapy, one patient declined adjuvant radiotherapy, and one patient’s prognosis indicated that the patient would no longer benefit from the radiotherapy.

A total of 14 (63.6%) out of 22 patients received adjuvant radiotherapy. Out of these 14 patients, six (42.9%) patients received only protons, five (35.7%) patients received only photons, and the remaining three (21.4%) patients received a combination of protons and photons.

The median follow-up period after initial diagnosis was 5 years, with a range from 0.2 to 10.5 years.

Of the 22 patients, six died during the follow-up period, including four within the first five years of follow-up. Radiological progression occurred in four patients.

**Fig. 1:**
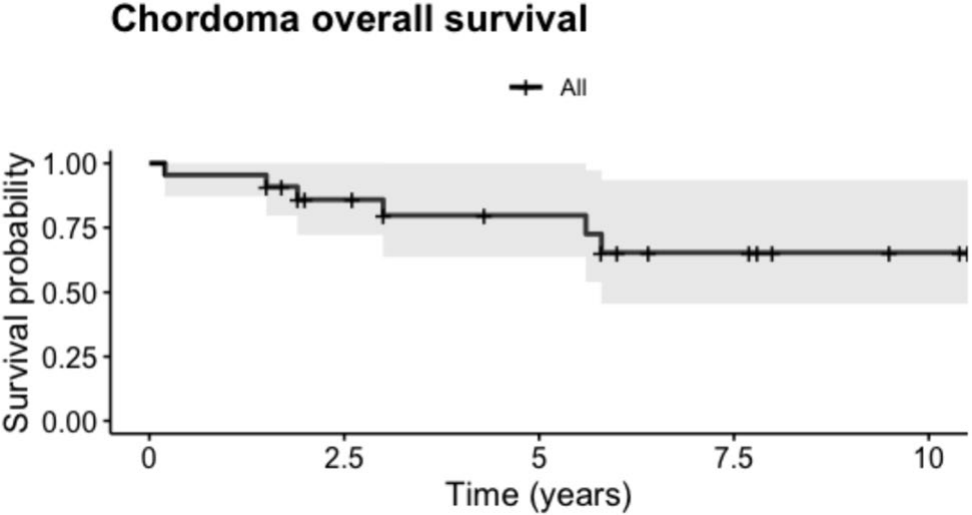
Kaplan–Meier survival analysis depicting overall survival for chordoma patients with 95% confidence intervals (grey shading)

**Fig. 2:**
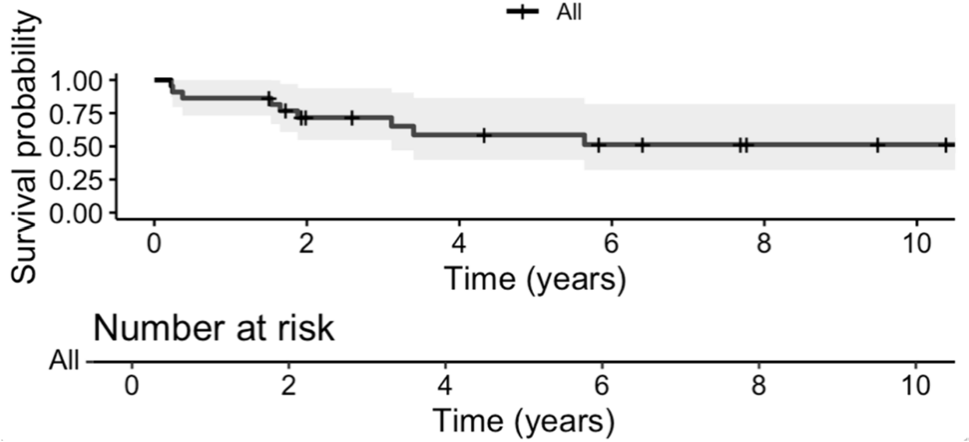
Kaplan–Meier survival analysis depicting progression-free survival for chordoma patients with 95% confidence intervals (grey shading)

The Kaplan–Meier plots showed a five-year overall survival rate of 79.1% (95% CI: 62.4—100) (Fig. 1) and a five-year progression-free survival of 58.6% (95% CI: 39.8 – 86.4) (Fig. 2).

**Fig. 3:**
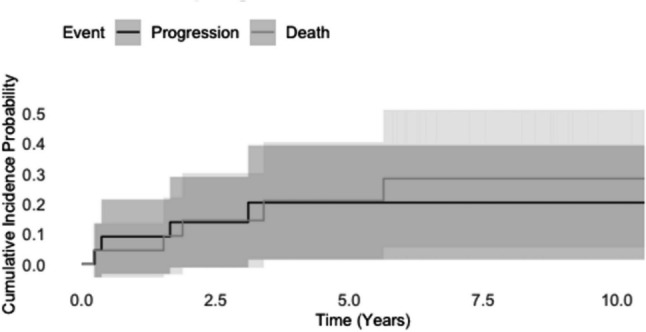
Competing risk analysis for chordoma patients. The plot illustrates cumulative incidence functions for the competing events: progression and death with 95% confidence intervals (grey shading)

The five-year cumulative incidence of progression in the competing risk analysis was 20.9% (95% CI: 3.1—39.1) (Fig. 3).

### Treatment modalities and survival rate for clival chondrosarcomas

Treatments and outcomes for patients with chondrosarcoma are summarized in Table 3.

**Table 3:** Treatment and outcome summary for clival chondrosarcoma patients

ID	Sex	Age at radiological diagnosis (years)	WHO Grade	Tumor volume on last pre-operative scan (cm^3^)	Surgical approach	Resection grade at index operation	Adjuvant radiotherapy	Number of surgical procedures	Alive at the time of data collection	Observation time (years)
2	Female	15	II	7.7	Trans-nasal	Partial	Yes	2	Yes	9.0
3	Female	24	I	5.6	Trans-nasal	Partial	Yes	3	Yes	5.7
4	Female	65	II	15.6	Trans-petrous	Partial	No	2*	No	0.4
7	Male	58	II	9.3	Subtemporal	GTR	No	2	Yes	9.4
13	Female	76	II	32.6	Subtemporal	STR	No	2	Yes	3.0
17	Female	42	I	10.6	OZ	Partial	No	2	Yes	6.8
18	Male	74	I	80.1	Trans-nasal	Partial	No	1	Yes	5.6
19	Female	40	II	27.6	Subtemporal	Partial	Yes	3	Yes	6.9
29	Male	29	II	10.1	OZ	GTR	No	2	Yes	4.6
34	Female	61	II	13.8	Subtemporal	GTR	No	2	Yes	0.0
35	Male	46	II	32.4	Trans-nasal	GTR	No	5	Yes	4.3

*Abbreviations*: *OZ* Orbitozygomatic approach, *STR* subtotal resection, *GTR* gross total resection

^*^ = Staged surgery

Regarding patients with chondrosarcomas, the mean age was 48.2 years, ranging from 15 to 76 years. At the time of diagnosis, the mean tumor volume was 22.3 cm^3^. The chondrosarcomas were histologically graded to be WHO II in 8 (72.7%) cases, while the remaining three (27.3%) cases were graded as WHO I. All patients underwent surgical resection. The trans-nasal and the subtemporal approach were the two most used approaches at the index surgery and was each applied in four (36.4%) cases. Among the four patients who underwent an index trans-nasal operation, one patient (25%) experienced cerebrospinal fluid leak as a complication.

Following the index operation, GTR was achieved in four patients (36.4%), STR in one patient (9.1%), partial resection in 6 patients (54.5%). 10 patients (90.9%) underwent multiple operations.

**Fig. 4:**
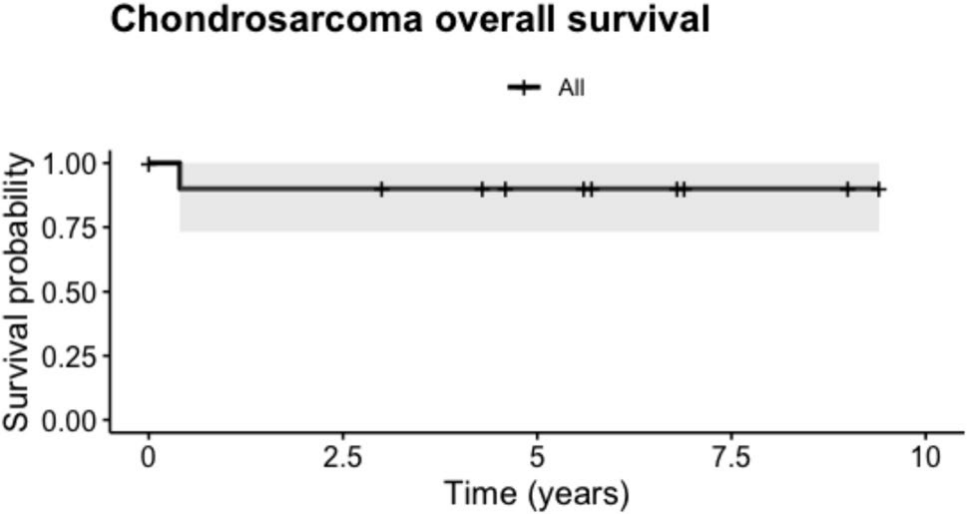
Kaplan–Meier survival analysis depicting overall survival for chondrosarcoma patients with 95% confidence intervals (grey shading)

**Fig. 5:**
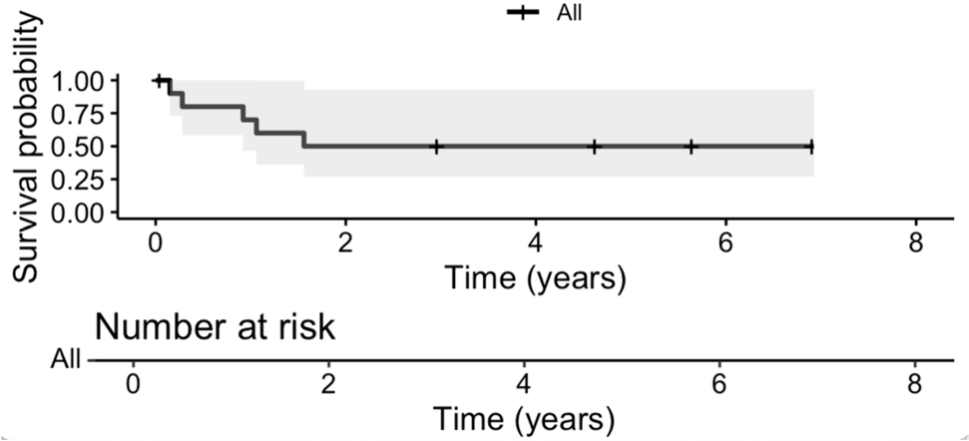
Kaplan–Meier survival analysis depicting progression-free survival for chondrosarcoma patients with 95% confidence intervals (grey shading)

Subsequently, three patients (27.2%) out of 11 patients received proton radiotherapy. All but one patient was alive at the time of follow-up. Radiological progression occurred in six patients. The median follow-up duration was 5.6 years, ranging from 0.0 to 9.4 years. The five-year overall survival rate was 90% (95% CI: 73.2—100;) (Fig. 4). The five-year progression-free survival was 0.50% (95% CI 26.9—92.9) (Fig. 5).

**Fig. 6:**
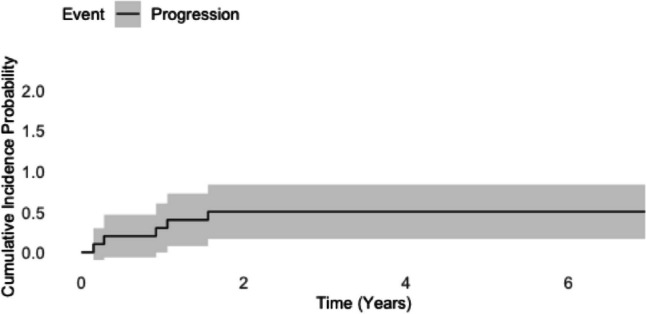
Competing risk analysis for chondrosarcoma patients. The plot illustrates a cumulative incidence function for the progression with 95% confidence intervals (grey shading). There were no competing events observed during the follow-up period

The five-year cumulative incidence of progression in the competing risk analysis was 50,0% (95% CI: 16.5—83.4) (Fig. 6).

### Postoperative complication

Table 4 details the complications regarding the initial surgery for both chordoma and chondrosarcoma. The number of patients with complications was 17 (51.5%). The most common complication observed after primary surgery was cranial nerve deficits, which were noted in 24.2% of patients. The cranial nerves affected included the abducens (n = 3), oculomotor (n = 2), trigeminal (n = 2), hypoglossal (n = 2), and vestibulocochlear (n = 1) nerves. The second largest complication category was infections, which occurred in 9.1% of patients.

**Table 4:** Complication rates for the initial operation

Complication type	Rate (%)
Infection	9.1
Cerebrospinal fluid leak	6.1
Cranial nerve deficit	24.2
Hemorrhage	6.1
Infarction	6.1
Pituitary dysfunction	3.0
Hydrocephalus	0.0
Other	0.0
Number of patients with complications	51.5

Patients who underwent the initial operation in the first half of the study period experienced a complication rate of 50.0% (95% CI: 25.4—74.6). Those who underwent their initial operation in the latter half of the study period had a complication rate of 57.1% (95% CI: 36.5—75.5). Fisher's Exact Test was conducted to compare the two groups, revealing no significant difference with a p-value of 0.73.

### Side effects to adjuvant radiotherapy

A single patient had radiotherapy-induced necrosis in the temporal lobe, which was diagnosed seven weeks after the completion of radiotherapy treatment. This complication caused epilepsy. Apart from this, no major complications were recorded. The most common side effects were fatigue (47.1%), followed by headache (41.1%) and nausea (35.3%). An overview of the symptoms ascribed to the adjuvant radiotherapy can be seen in Table 5.

**Table 5:** Symptoms that can be ascribed to the adjuvant radiotherapy for patients with clival chordoma or chondrosarcoma

ID	Sex	Age at radiological diagnosis (years)	Histological diagnosis	Treatment modality	Symptoms that can be ascribed to radiation therapy	Objective findings that can be ascribed to radiation therapy
14	Male	35	Chordoma	Proton, photons	Headache, nausea, fatigue	-
24	Male	61	Chordoma	Photons	Fatigue	-
2	Female	15	Chondrosarcoma	Protons	Headache, fatigue, Epilepsy	Necrosis in the temporal lobe
3	Female	24	Chondrosarcoma	Protons	Headache, hearing problems	-
19	Female	40	Chondrosarcoma	Protons	Dizziness	Transitory temporal and/or frontotemporal alopecia
12	Female	49	Chordoma	Protons	Dizziness, nausea	-
15	Female	60	Chordoma	Photons	Change in sense of smell	-
20	Male	40	Chordoma	Photons	Neck pain, nausea, fatigue	-
21	Female	51	Chordoma	Photons	Hearing problems, neck pain, headache, nausea, fatigue	Hearing loss
22	Male	54	Chordoma	Protons	-	-
23	Male	54	Chordoma	Protons	Headache	-
25	Male	64	Chordoma	Photons	-	-
30	Male	36	Chordoma	Protons	Hearing problems, neck pain, headache, nausea, fatigue	-
31	Male	34	Chordoma	Protons, photons	Hearing problems, dysfonia, neck pain, nausea	Dysphonia
32	Male	80	Chordoma	Protons	-	-
33	Male	70	Chordoma	Protons	Fatigue	-
36	Female	68	Chordoma	Protons, photons	Headache, fatigue	-

## Discussion

In the present national consecutive series, the five-year overall survival rate for patients with chordoma was 79.1% (95% CI: 62.4–100), and 63.6% of these patients received adjuvant radiotherapy.

The five-year overall survival for patients with chondrosarcoma was 90% (95% CI: 73.2–100), and 27.2% of them received adjuvant radiotherapy. The complication rate for the index surgery was 51.5%.

While numerous analogous case series exist, differences in scope and methods make direct international comparisons challenging. This is especially true concerning clival chondrosarcomas since they are often grouped with chondrosarcomas in other locations in the skull or skull base or in the upper cervical region.

In this national series, as in other similar studies, clival chondrosarcoma was rarer than clival chordoma. The per-million-person-years incidence rates were estimated to be 0.30 for clival chordoma and 0.16 for clival chondrosarcoma, which are close to the combined incidence rate of 0.3 reported in the literature [2, 31].

Metcalfe et al. (2021) [19] provide a detailed account of a nine-year (2007–2016) consecutive case series with 24 patients from a UK tertiary referral center who underwent treatment for skull base chondrosarcomas or clival chordomas. Not all chondrosarcomas were situated in the clivus; some were merely adjacent, arising from different bone structures. Distinctive differences in treatment modalities were observed relative to our series. In their series, 21 of 24 (87.5%) patients received adjuvant radiotherapy. All clival chordomas in Metcalfe’s series were treated using trans-nasal endoscopic resection. In contrast, in our study, 18 of the 33 (54.5%) initial surgeries were executed transnasally [19].

Direct comparisons concerning operative complications are difficult to make, as case studies present complications in different degrees of detail. However, for some types of complications, a direct comparison can be made, and in general, the complication rate in our study was within the rate interval reported by other case studies.

Bai et al. (2022) presents a large retrospective study from China that included 284 patients with clival chordoma. The most common surgical complications were cranial nerve injury (7.9%) followed by CSF leak (3.9%).

Passeri et al. (2022) [22] presents a retrospectively study that examined 210 patients treated in France between 1991 and 2020 for clival and craniovertebral junction chordomas. The surgical complication rate cranial nerve deficits were 17.7% and the rate for CSF leak was 12.1%. The risk of complications is weighed against the potential benefits of aggressive tumor resection.

In our case series, GTR was achieved in 31.8% of cases, somewhat lower than Bai et al. and Passeri et al., who reported GTR rates of 40.1% and 43.8%, respectively.

The complication rates vary between case series, possibly due to differences in methodology for recording complications or the correlation between surgical expertise and complication rates. The most common surgical complications were cranial nerve deficit followed by CSF leak. The complication rate for cranial nerve deficits in our series was general higher than shown in other case [3, 12, 14, 22] while the rate of CSF leak where similar [3, 14, 19, 22]. However, according to a literature review, up to 80% of patients exhibit new cranial nerve deficits following the resection of skull base chordomas and chondrosarcomas [16].

When considering surgical complications in general, the results from other case series were like ours. Förander et al. reported a complication rate of 33% for the index operation [12].

In Jägersberg et al. [14], 53.8% of patients had at least one surgical complication.

One of the primary arguments for centralizing specialized treatments at a single center is that it allows surgeons to gain experience, potentially reducing complication rates. However, our study did not establish such a correlation, likely due to the small sample sizes involved.

The most common side effects of radiotherapy to the skull base observed in this series are well-known acute toxicities due to edema in the irradiated normal tissue. Headaches and dizziness during and in the weeks following irradiation respond well to prednisolone treatment and are usually self-limiting. Cranial nerves that are affected by the tumor or damaged by surgery are at a higher risk of developing persistent neuropathies after adjuvant radiotherapy. The radiation dose to the optic chiasm and the brainstem is, in most cases, dose-limiting, and compromises based on the coverage of the residual tumor and surgical cavity are often necessary. In this series, no cases of vision loss have been reported, suggesting that the utilized constraints are effective.

A 2015 consensus paper on clinical practice guidelines for chordoma treatment recommended offering adjuvant radiotherapy to all chordoma patients following macroscopic complete surgery. However, the evidence level for this recommendation is low, and the clinical benefit is described as limited [25]. This recommendation is supported by Passeri et al. (2022) [22] who found that postoperative radiation therapy improved progression-free survival but not overall survival [22]. Jägersberg et al. (2017) [14] presented a retrospective report on 13 patients with clival chordomas treated in Switzerland between 2005 and 2015. All patients underwent radiotherapy after surgery. The observed five-year overall survival rate was 83% [14].

Förander et al. (2017) [12] presented a similar retrospective case series of 22 patients diagnosed with and treated for clival chordomas in Sweden from 1984 to 2015. All patients underwent radiotherapy after surgery. The reported five-year overall survival was 82% [12].

Compared to the similar studies the use of adjuvant radiotherapy was more limited in our case series. There was no routine referral for radiotherapy in patients without evidence of tumor remnants on follow-up MRI. We found no indication that this affected five-year survival rates, which in our case series were consistent with the results of other studies [10, 12, 14, 19, 21, 22, 24, 25, 30]. However, we cannot rule out that this approach to adjuvant radiotherapy could impact survival rates over a longer follow-up period.

### Limitations and strengths of the study

The present study shares the common constraints of retrospective observational studies. In such studies, various individual treatment decisions, which are driven by the prevailing state of knowledge and a commitment to delivering optimal care to individual patients, often lack coordination. The absence of standardized treatment protocols and the limited size of study samples render many statistical comparisons and assessments imprecise. Nevertheless, it remains feasible to assess whether our findings and observations are aligned with established treatment guidelines and prevailing medical beliefs. Furthermore, it is important to consider robust and objective endpoints in our analysis, such as five-year overall survival. Unfortunately, due to limited access, we were unable to provide precise details regarding the causes of death for some of our patients. Consequently, we cannot dismiss the possibility that these patients may have succumbed to causes of death that were unrelated to their tumors. The case series presents outcomes for all occurrences within a single country, and we confirmed the accuracy of the histological diagnoses using pathological data. Merging survival data can create an excessively optimistic perception of tumor control rates because low-grade chondrosarcomas typically exhibit a less aggressive pattern. [2, 11, 18]

## Conclusion

The overall results, as indicated by the incidence, complication, and five-year overall survival rates are consistent with comparable case series. The follow-up period is, however, too short to draw any firm conclusions.

Echoing findings derived from prior series, clival chordomas showed poorer prognoses than chondrosarcomas. The available case series are difficult to generalize and draw conclusions from due to disparities in treatment modalities and tumor localization. The main difference between this national cohort and the other series is the former’s less rigorous use of adjuvant radiotherapy, especially for chordomas. This should not be considered an argument for a less rigorous use of radiation, as earlier studies have established a positive effect of adjuvant radiation therapy on progression-free survival [22, 25].

Due to the rarity of these tumors and the involvement of multiple treatment modalities, it is essential to discuss patient management in a multidisciplinary setting, and further studies, ideally with the pooling of international data, are crucial to ensure optimal treatment pathways.

## Data Availability

The patient data was collected from the 'Sundhedsplatformen' and stored in a REDCap database. The data were extracted, anonymized, and organized into an Excel spreadsheet for the creation of tables. Access to the anonymized data can be requested by contacting the corresponding and first author, Mikkel Skotting.

## Code availability

The code for generating Kaplan–Meier plots is available upon request by contacting Mikkel Skotting, the corresponding and first author.
